# Giant Right Atrial Aneurysm: Observation Versus Intervention

**DOI:** 10.1016/j.jaccas.2026.109192

**Published:** 2026-07-09

**Authors:** Nabil Alhayek, Mohammad Osama, Harold M. Burkhart, Mohanageetha Ardhanari, Arshid Mir, Andrew Cave

**Affiliations:** a Department of Pediatrics, University of Oklahoma Health Sciences Center, Oklahoma City, Oklahoma, USA; b University of Oklahoma Health Sciences Center, College of Medicine, Oklahoma City, Oklahoma, USA; c Department of Surgery, University of Oklahoma Health Sciences Center, Oklahoma City, Oklahoma, USA; d Section of Pediatric Cardiology, University of Oklahoma Health Sciences Center, Oklahoma City, Oklahoma, USA

**Keywords:** atrial thrombus, congenital heart disease, dchs1 mutation, right atrial aneurysm

## Abstract

**Background:**

Idiopathic right atrial aneurysm (IRAA) is rarely reported in pediatric literature. Clinical presentations range from incidental findings to serious complications including arrhythmias, thrombosis, and cardiac arrest.

**Case Summary:**

A 4-year-old girl with DCHS1 mutation, autism spectrum disorder, and congenital IRAA managed conservatively presented to the emergency department with 3-day history of viral symptoms. Shortly after arrival, the patient developed unstable atrial flutter requiring electric cardioversion. Later, imaging demonstrated a 1.2 × 1.2-cm right atrial thrombus in a massively dilated right atrium. The patient underwent successful surgical atrial reduction and thrombectomy.

**Discussion:**

IRAA has been rarely reported in pediatric literature. Half of the reported cases were diagnosed incidentally. Our case is unique in its rapid progression to serious complications despite recently initiated aspirin therapy.

**Take-Home Messages:**

This case proposes a possible association between DCHS1 variants and IRAA and highlights the importance of considering prophylactic surgical intervention in select pediatric patients to prevent serious complications.

## Visual Summary

**Figure:**
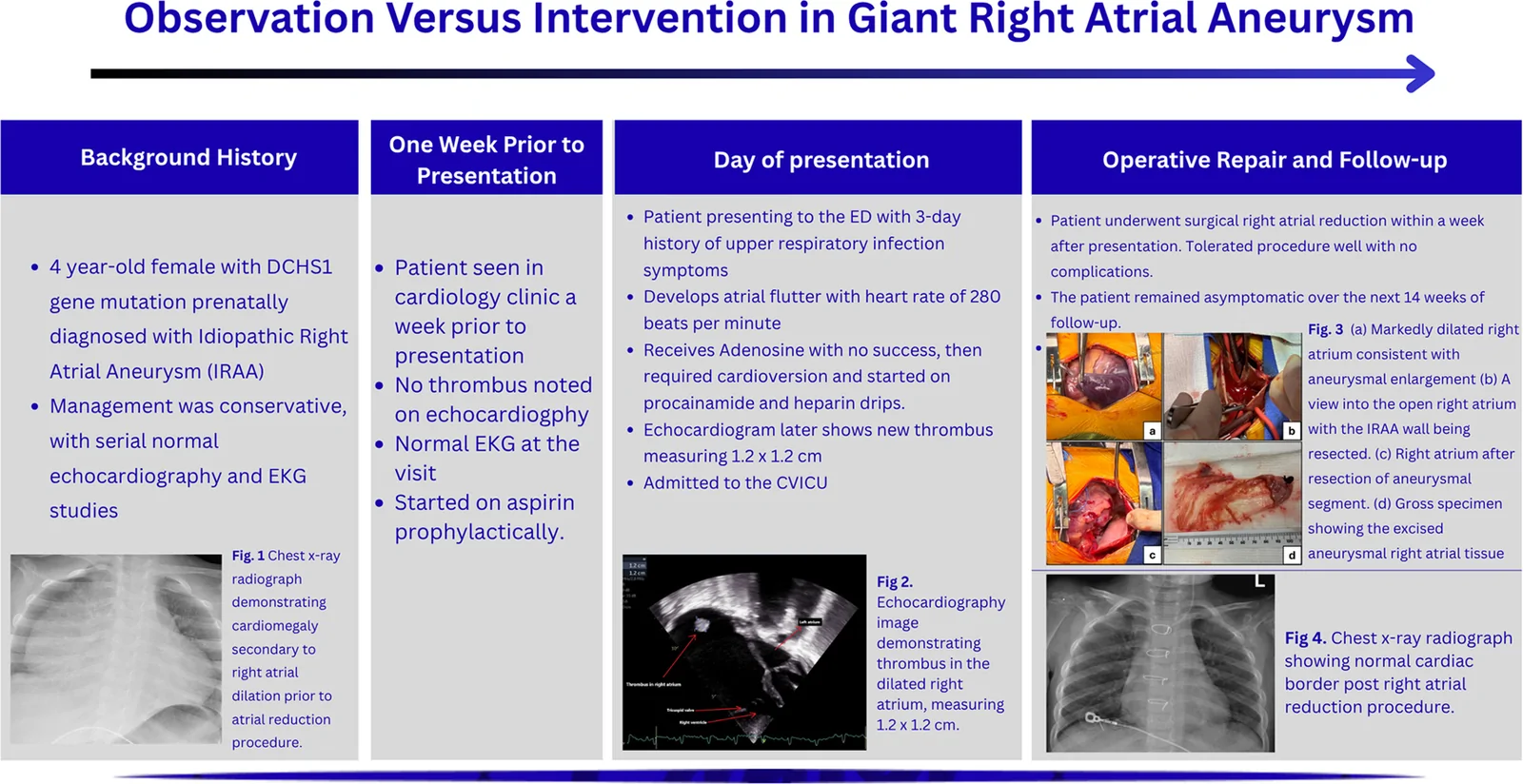

**Visual Summary:**
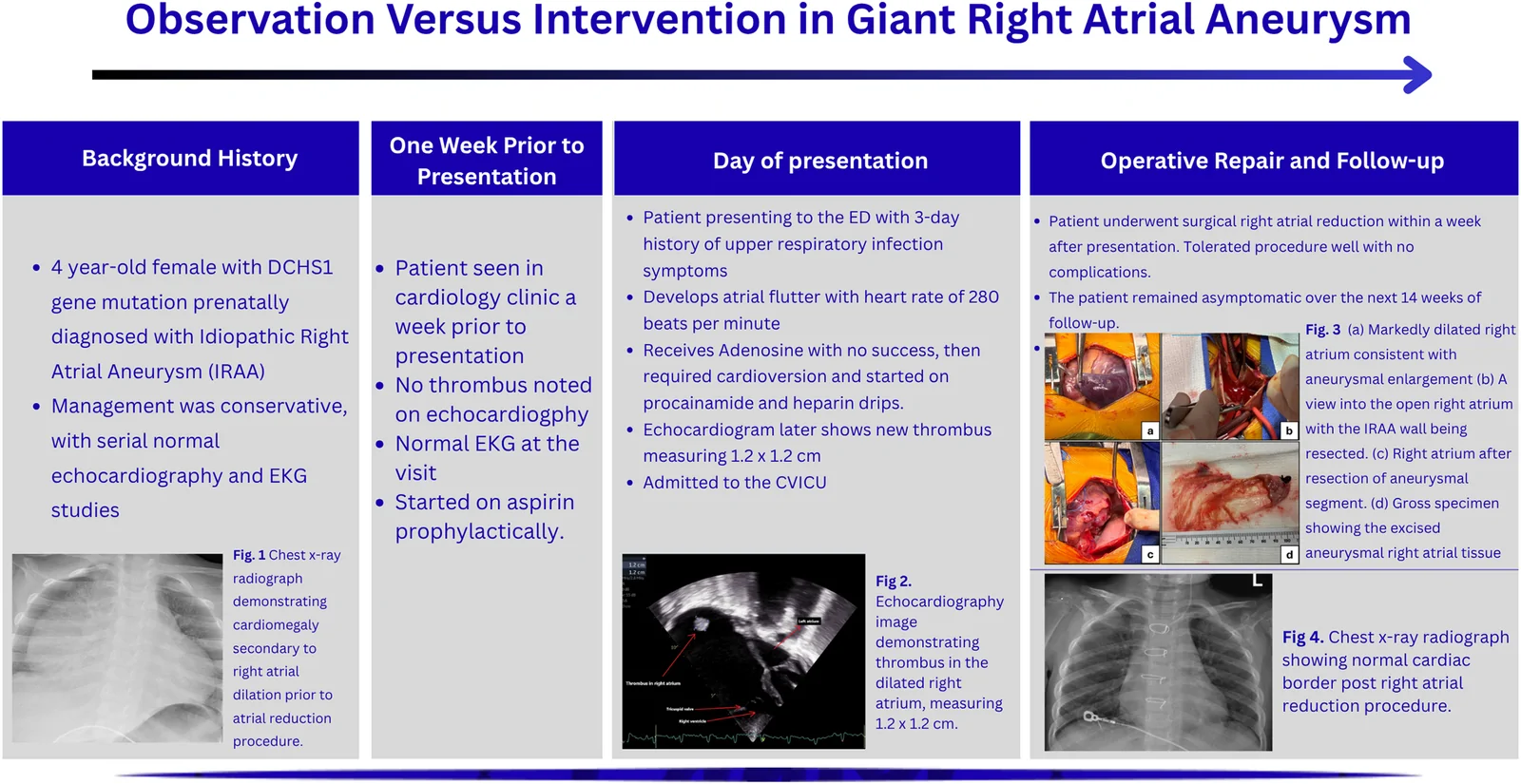
Case Illustration Demonstrating the Timeline of the Case Starting With a Background History, followed by the Patient's Clinical Course Leading to the Surgical Repair of their Right Atrial Aneurysm CVICU = cardiovascular intensive care unit; ED = emergency department; EKG = electrocardiogram.

## History of Presentation

A 4-year-old girl with known idiopathic right atrial enlargement, DCHS1 gene mutation, and autism presented to the emergency department with a 3-day history of vomiting, congestion, and diarrhea. The patient was afebrile, with a normal heart rate of 98 beats/min, and vitals were stable at the time. The patient was diagnosed with idiopathic right atrial aneurysm (IRAA) prenatally when an abnormal fetal echocardiography revealed a massively dilated right atrium. Repeating echocardiography at 24 hours of life confirmed the diagnosis. The patient remained completely asymptomatic with normal biventricular functions and was discharged home with close cardiology monitoring. Serial electrocardiography, echocardiography, and Holter monitoring remained stable across multiple follow-up visits, during which conservative management was pursued.

**Take-Home Messages**

• This case proposes a possible association between DCHS1 variants and IRAA.
• Close monitoring for patients with IRAA is necessary despite absence of symptoms given possibility for rapid, sudden development of complications.
• Complications of IRAA include arrhythmias, right atrial thrombosis, cardiac arrest or rupture. Prophylactic surgical intervention in select pediatric patients might be indicated to prevent such serious complications.

The patient had been seen just a week before their emergency department presentation for routine surveillance, at which time they were asymptomatic. Echocardiography at that visit showed a severely dilated right atrium, no thrombus present, trace mitral valve regurgitation, and normal biventricular function. A screening Holter monitor was pending, and the patient was prescribed 81 mg of aspirin for thromboprophylaxis.

## Past Medical History

The patient's medical history included congenital IRAA, autism spectrum disorder, and DHCS1 gene mutation.

## Differential Diagnosis

Viral gastroenteritis, upper respiratory infection, pulmonary embolism, arrhythmia, ST-elevated myocardial infarction.

## Investigations

**Figure 1:**
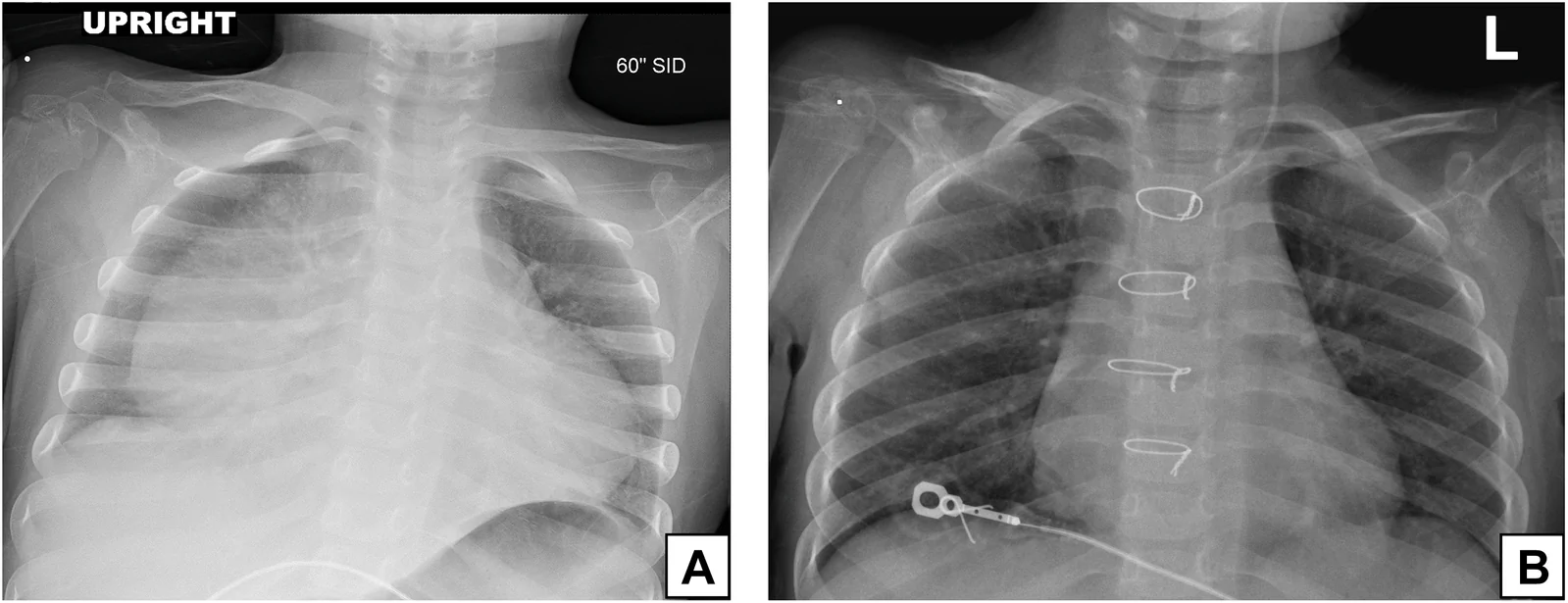
Chest X-Ray Pre- and Post-Atrial Reduction (A) Chest x-ray demonstrating cardiomegaly secondary to right atrial dilation before atrial reduction procedure. (B) Chest x-ray showing normal cardiac border post right atrial reduction procedure.

Respiratory viral testing was positive for coronavirus. Chest x-ray showed cardiomegaly, stable in size compared with prior images (Figure 1). While sleeping, the patient developed tachycardia, with their heart rate rising to 280 beats/min as observed on pulse oximetry. A 0.2-mg/kg dose of intravenous adenosine was administered, revealing an underlying rhythm of atrial flutter on electrocardiogram.

## Management

**Figure 2:**
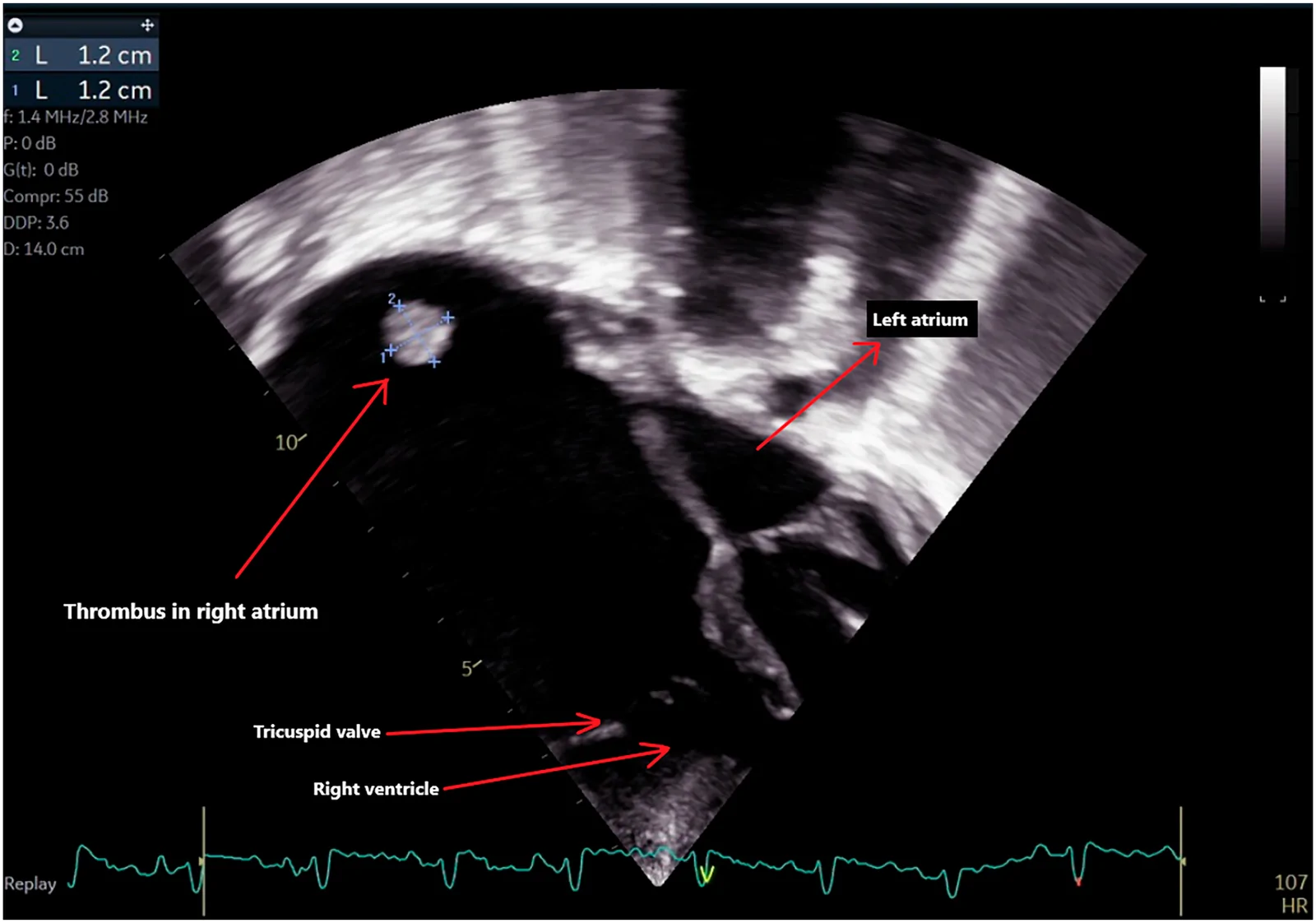
Echocardiography Image Demonstrating Thrombus in the Dilated Right Atrium, Measuring 1.2 × 1.2 cm

**Figure 3:**
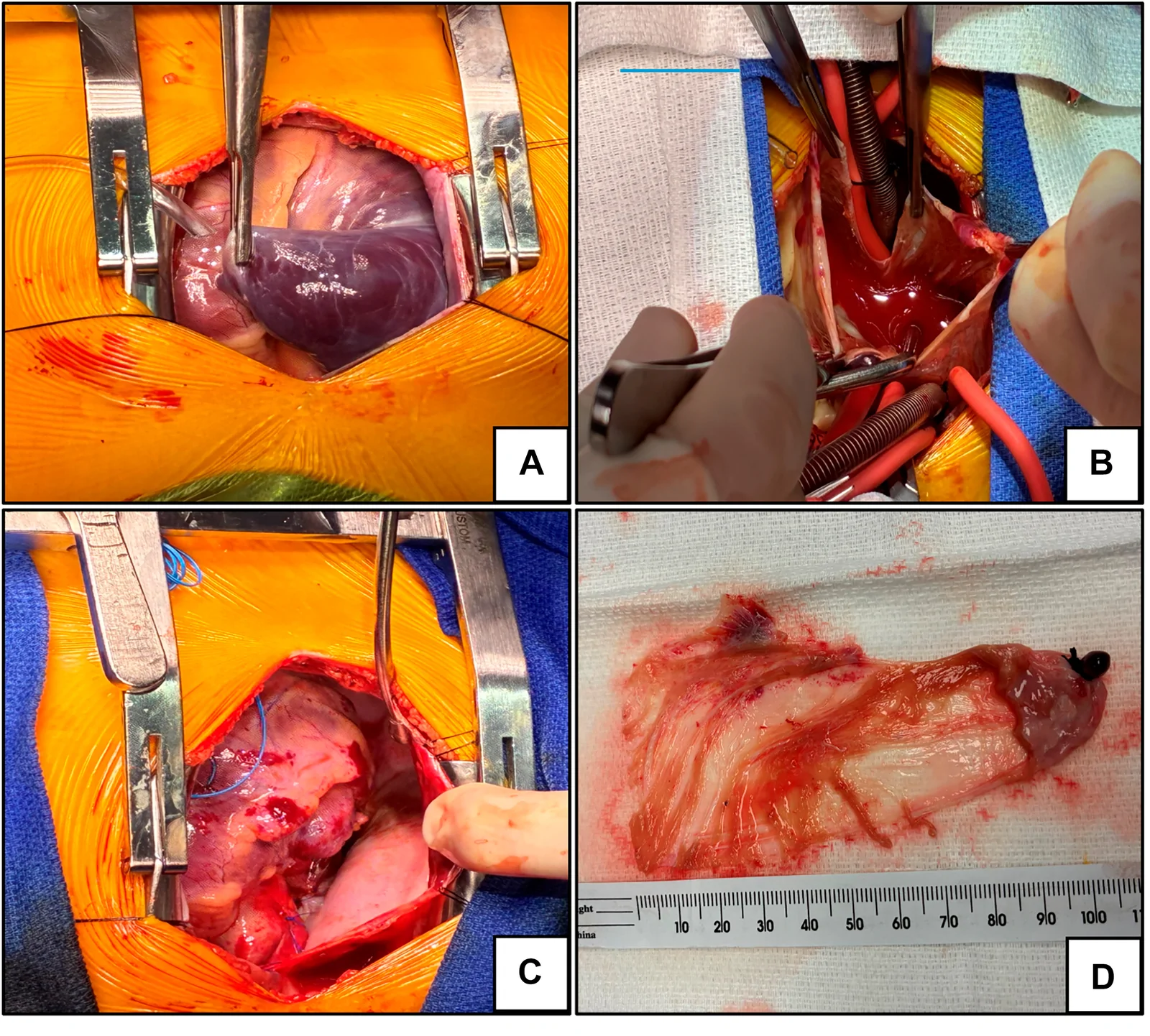
Intraoperative Findings and Surgical Management of Idiopathic Right Atrial Aneurysm (A) Initial exposure demonstrates a markedly dilated, thin-walled right atrium consistent with aneurysmal enlargement. (B) A view into the open right atrium with the idiopathic right atrial aneurysm wall being resected. (C) View of right atrium after resection of aneurysmal segment. (D) Gross specimen showing the excised aneurysmal right atrial tissue.

Because of the extremely rapid heart rate, synchronized electrical cardioversion was performed. The patient successfully converted to sinus rhythm with a heart rate in the low 100s. A procainamide drip and therapeutic heparin drip were started, and the patient was transferred to the cardiovascular intensive care unit. A transthoracic echocardiography study performed later during the day demonstrated a new 1.2 × 1.2-cm thrombus in the right atrium (Figure 2) with preserved biventricular systolic function. One week after presentation to the emergency department, the patient underwent right atrial aneurysm reduction (Figure 3). The atrium was excised, leaving a 2-cm cuff along the atrioventricular groove. The incision was carried toward the interatrial septum and then extended across the atrial appendage, leaving the sinus node intact. The patent foramen ovale was closed, and the atrial thrombus was removed. The patient tolerated the procedure well, with an uncomplicated postoperative course.

## Outcome and Follow-Up

Echocardiography before discharge showed a mildly dilated right atrium, trace tricuspid valve regurgitation, and normal biventricular systolic function. The patient was managed postoperatively with milrinone, nicardipine, and procainamide, and later discharged home on aspirin 81 mg and flecainide. The patient remained asymptomatic over the next 14 weeks of follow-up.

## Discussion

IRAA is rare, with fewer than 70 cases reported across pediatric populations.^1^ Approximately half of the reported cases were asymptomatic and diagnosed incidentally on radiographs obtained for unrelated reasons, whereas the remaining patients presented with more serious features including arrhythmias, dyspnea, and palpitations.^1^ Reported complications include right atrial thrombosis, compression of adjacent structures, and rupture.^2^^,^^3^

Our case is unique in its rapid progression to serious complications, including atrial flutter and atrial thrombus, despite the patient being completely asymptomatic, on prophylactic daily aspirin, and without evidence of a thrombus on echocardiography obtained only 1 week before presentation. Our patient also carries a DCHS1 gene mutation, a gene encoding a protocadherin involved in cardiac structural integrity; however, although DCHS1 mutations have been associated with mitral valve prolapse and van Maldergem syndrome,^4^^,^^5^ an association with IRAA has not been previously reported to our knowledge. DCHS1 deficiency has been shown to cause altered cellular migration and patterning in valve interstitial cells,^4^ suggesting a potential mechanism by which DCHS1 variants could contribute to atrial structural abnormalities. Whether the co-occurrence of DCHS1 mutation and IRAA in our patient represents a genotype-phenotype correlation or coincidental findings warrants further investigation.

The large size of these aneurysms and the relative absence of pectinate muscles may result in sluggish blood flow within the dilated atrial chamber, predisposing patients to thrombus formation.^6^ In addition, atrial stretching and structural remodeling may disrupt normal conduction pathways, increasing vulnerability to reentrant circuits and ectopic activity, thereby predisposing patients to atrial arrhythmias.^7^ Adverse outcomes such as thrombosis and arrhythmias have been associated with risk factors including large right atrial or atrial appendage size, as well as the presence of concomitant congenital heart defects.^1^

Management options of IRAA include surgical resection, conservative medical therapy with follow-up, or observation without intervention.^1^ Surgical resection is recommended in symptomatic patients; however, the optimal management of asymptomatic IRAA remains controversial. Some authors advocate for prophylactic surgical resection to avoid serious complications^8^ and others recommend conservative management.^4^ A systematic review by Zhang et al^1^ including 153 patients with IRAA (70 were pediatric patients) found that conservative management was associated with poorer outcomes; however, most conservatively managed patients were symptomatic at diagnosis (58%), limiting the ability to extrapolate these findings to asymptomatic patients. Given the outcome observed in the present case and similar reports in the literature, prophylactic surgical intervention might be preferable in select asymptomatic patients to reduce the risk of developing life-threatening complications. We suggest consideration of elective surgical resection of IRAA in asymptomatic patients based on the presence of 1 or more of the following criteria: 1) significant right atrial enlargement, defined by absolute size or relative chamber comparison (eg, right atrial volume index or disproportionate enlargement relative to the right ventricle or combined left-sided chambers); 2) documented atrial arrhythmias on routine screening; and 3) evidence of intracardiac thrombus on screening echocardiography. Despite increasing recognition of IRAA, standardized management guidelines remain lacking because of the rarity of the condition. Decisions regarding timing and indication for surgical intervention should therefore be individualized and guided by clinical presentation, imaging findings, and multidisciplinary discussion. Longitudinal follow-up and accumulation of additional case reports and series are needed to better define risk stratification and establish evidence-based recommendations for the management of asymptomatic patients.

## Conclusions

IRAA is an uncommon cardiac anomaly in children. Although the literature does not currently clearly establish whether surgical management is superior to conservative treatment in asymptomatic patients with IRAA, multiple reports, including the case presented here, demonstrate that surgery can be performed safely and effectively. Surgical management may reduce associated complications related to arrhythmias and thromboembolism. Our case underscores the importance of considering prophylactic surgical intervention in select pediatric patients with IRAA to reduce the risk of arrhythmia and thrombus formation.

## Funding Support and Author Disclosures

The authors have reported that they have no relationships relevant to the contents of this paper to disclose.
